# Longitudinal genome-wide association analysis using a single-step random regression model for height in Japanese Holstein cattle

**DOI:** 10.3168/jdsc.2022-0347

**Published:** 2023-07-13

**Authors:** Toshimi Baba, Gota Morota, Junpei Kawakami, Yusaku Gotoh, Taro Oka, Yutaka Masuda, Luiz F. Brito, Rebbeca R. Cockrum, Takayoshi Kawahara

**Affiliations:** a Holstein Cattle Association of Japan, Hokkaido Branch, Sapporo, Hokkaido, Japan 001-8555; b School of Animal Sciences, Virginia Polytechnic Institute and State University, Blacksburg, VA 24061; c Holstein Cattle Association of Japan, Tokyo, Japan 164-0012; d Department of Sustainable Agriculture, Rakuno Gakuen University, Ebetsu, Hokkaido, Japan 069-8501; e Department of Animal Sciences, Purdue University, West Lafayette, IN 47907

## Abstract

Growth traits, such as body weight and height, are essential in the design of genetic improvement programs of dairy cattle due to their relationship with feeding efficiency, longevity, and health. We investigated genomic regions influencing height across growth stages in Japanese Holstein cattle using a single-step random regression model. We used 72,921 records from birth to 60 mo of age with 4,111 animals born between 2000 and 2016. The analysis included 1,410 genotyped animals with 35,319 single nucleotide polymorphisms, consisting of 883 females with records and 527 bulls, and 30,745 animals with pedigree information. A single genomic region at the 58.4 megabase pair on chromosome 18 was consistently identified across 6 age points of 10, 20, 30, 40, 50, and 60 mo after multiple testing corrections for the significance threshold. Twelve candidate genes, previously reported for longevity and gestation length, were found near the identified genomic region. Another location near the identified region was also previously associated with body conformation, fertility, and calving difficulty. Functional Gene Ontology enrichment analysis suggested that the candidate genes regulate dephosphorylation and phosphatase activity. Our findings show that further study of the identified candidate genes will contribute to a better understanding of the genetic basis of height in Japanese Holstein cattle.

## Highlights

• Growth traits are economically important in dairy cattle.
• A longitudinal genome-wide association study was applied to height.
• A single genomic region on chromosome 18 consistently showed a significant effect on height over time.
• The identified candidate gene may have a pleiotropic effect on various important traits in dairy cattle.

## Graphical Abstract

**Figure:**
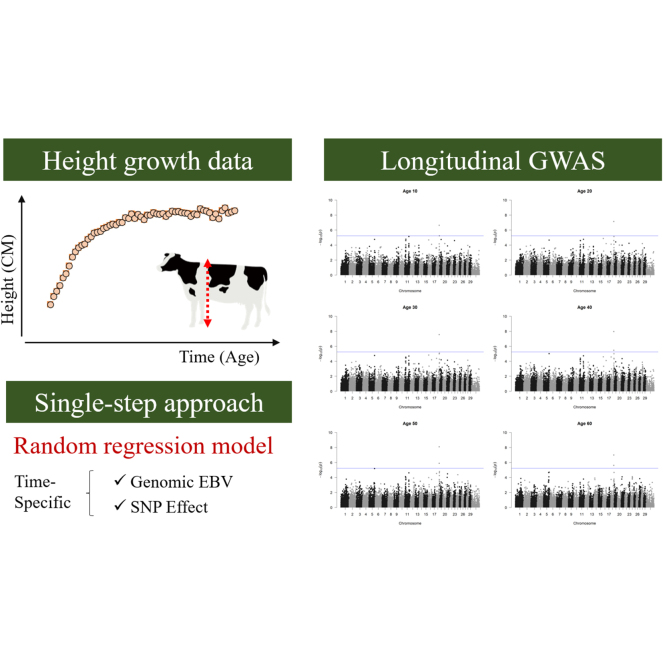
**Summary:** A random regression model is capable of modeling traits that are repeatedly measured at multiple time points. This study aimed to dissect the genetic change of height over time in Japanese Holsteins using a longitudinal genome-wide association approach. We identified a single genomic region on chromosome 18, which consistently showed a significant effect on height. This study supports the importance of the identified genomic region because it is also reported to influence body conformation, longevity, and calving traits in past studies.

Growth traits such as BW and height play an economically important role in reducing feeding costs and enhancing the profit of dairy farms. Poor growth during the rearing period leads to a delay in age at first calving and increases culling risk at an earlier age. In addition, heifer growth rates are related to milk production during the first lactation (Van De Stroet et al., 2016). Previous studies have also reported moderate-to-high heritability estimates for growth traits (Gallo et al., 2001; Brotherstone et al., 2007; Yin and König, 2018).

Growth traits are longitudinal records measured repeatedly at multiple time points. The random regression model (**RRM**) is applied to the genetic analysis of growth traits because it captures genetic changes (i.e., genetic correlation) across time by fitting the additive genetic effect as a covariance function (Mrode, 2014). Previous studies have also reported that genetic correlations among age groups are not constant for growth traits (Arango et al., 2004; Brotherstone et al., 2007; Yin and König, 2018), indicating that the use of RRM may lead to more accurate predictions than simpler statistical models, such as repeatability, which assumes a constant genetic correlation across growth stages.

The RRM also provides a better insight into the genomic regions affecting time-dependent traits (Lund et al., 2008; Ning et al., 2017). The single-step genomic best linear unbiased prediction model accounts for genomic pre-selection, directly obtaining genomic enhanced breeding values by the simultaneous use of both pedigree and genomic information without involving additional steps (Aguilar et al., 2010; Patry and Ducrocq, 2011). The RRM based on the single-step genomic best linear unbiased prediction has been applied in several studies to evaluate longitudinal traits in dairy cattle (Koivula et al., 2015; Baba et al., 2017; Oliveira et al., 2019a). The single-step genome-wide association study (**ssGWAS**) method is an extension of the single-step genomic best linear unbiased prediction to the GWAS framework (Wang et al., 2012). Recently, ssGWAS has been used to explore the genome-to-phenome relationships in animals (Wang et al., 2014) and can be combined with RRM (Oliveira et al., 2019b; Buaban et al., 2022). For instance, Oliveira et al. (2019b) conducted ssGWAS for milk production traits in 3 dairy cattle breeds by leveraging multi-trait RRM and reported that the identified candidate genes varied across lactations and breeds. Although a previous study (Yin and König, 2019) found significant SNP at different ages for a direct genetic effect on BW in Holstein cattle, the use of RRM-ssGWAS to investigate the genetic basis of dairy cattle growth traits over time is limited.

Some countries include body size in their selection indices because it is genetically related to health, feed efficiency, and longevity (Miglior et al., 2017). Body size of Japanese Holstein females, measured in terms of BW and height, has been increasing recently (Kawakami et al., 2021). Although height is not yet included in the Japanese selection index, this suggests that controlling their body size via genetic selection might become an important breeding goal in the near future. As no GWAS of height has been reported in the Japanese Holstein cattle population, the objective of this study was to identify genomic regions influencing height across growth stages by leveraging the longitudinal ssGWAS approach.

Animal care and use approval was not required because all data were obtained from a pre-existing database.

Growth data for wither height were collected from a commercial dairy farm in Ebetsu, Hokkaido, and 10 national experimental dairy farms in the Niikappu and Iwate station at the National Livestock Breeding Center (Nishishirakawa, Fukushima), the Hokkaido Agricultural Research Center in National Agriculture and Food Research Organization (Sapporo, Hokkaido), the Agricultural Research Department in Hokkaido Research Organization (Nakashibetsu, Hokkaido), the Yamagata Integrated Agricultural Research Center (Yamagata, Yamagata), the Gunma Prefectural Livestock Experiment Station (Maebashi, Gunma), the Chiba Prefectural Livestock Experiment Station (Yachimata, Chiba), the Gifu Prefectural Livestock Research Institute (Gifu, Gifu), the Awaji Agricultural Technology Center (Minamiawaji, Hyogo), and the Nagasaki Agricultural and Forestry Technical Development Center (Isahaya, Nagasaki) in Japan. We included individuals born between 2000 and 2016 with at least 3 records from birth to 60 mo of age. The animals were required to have records before 10 and after 30 mo of age. All animals included in this study were registered by the Holstein Cattle Association of Japan (Nakano, Tokyo, Japan). Phenotypic records that deviated from the mean by more than 5 times the standard deviation at each age were excluded as outliers. After data editing, 72,921 records from 4,111 animals were retained for subsequent analyses. The average (± SD) number of records per animal was 17.7 ± 8.3.

We used 883 females with records and 527 bulls, which were either their sires or maternal grand-sires. These individuals were genotyped using the Illumina Bovine50 BeadChip or BovineLD BeadChip (Illumina), designed based on the UMD3.1 genome assembly, and missing genotypes were imputed using findhap.f90 software (VanRaden et al., 2011). A total of 35,319 SNP with minor allele frequency ≥0.05 and call rate ≥0.90 were retained for further analyses. In addition, 30,745 animals with pedigree information tracing back to 5 generations were also included.

The genomic enhanced breeding values (**GEBV**) of height for genotyped animals were estimated using RRM-ssGWAS using the following equation:

$$y_{ijlt}=HY_{i}+M_{j}+\overset{4}{\underset{k=0}{\sum }}\phi {\left(t\right)}_{lk}b_{k}+\overset{2}{\underset{k=0}{\sum }}\phi {\left(t\right)}_{lk}u_{lk}+\overset{2}{\underset{k=0}{\sum }}\phi {\left(t\right)}_{lk}pe_{lk}+e_{ijlt},$$

where *y_ijlt_* is the observation of the *l*th animal at age *t*, *HY_i_* is the fixed effect of *i*th herd-birth-year combination, *M_j_* is the fixed effect of *j*th birth-month, *b_k_* is the *k*th fixed regression coefficient explaining the overall mean, *u_lk_* is the *k*th random regression coefficient of additive genetic effect, *pe_lk_* is the *k*th random regression coefficient of permanent environment effect, *e_ijlt_* is the residual, and $\phi {\left(t\right)}_{lk}$ is the *k*th Legendre orthogonal polynomial of animal *l* evaluated at age *t*. In matrix notation, **y** = **Xb** + **Z**_1_**a** + **Z**_2_**p** + **e**, where **y** is the vector of phenotype, **b** is the vector of fixed effects, **a** is the vector of random additive genetic effect, **p** is the vector of random permanent effect, **e** is the vector of residuals, and **X**, **Z**_1_, and **Z**_2_ are incidence matrices. The corresponding mixed model equations (**MME**) can be shown as follows:

$$\begin{array}{l} \left[\begin{array}{ccc} X^{\prime }R^{-1}X & X^{\prime }R^{-1}Z_{1} & X^{\prime }R^{-1}Z_{2}\\ Z_{1}^{\prime }R^{-1}X & Z_{1}^{\prime }R^{-1}Z_{1}+V_{g}⊗H^{-1} & Z_{1}^{\prime }R^{-1}Z_{2}\\ Z_{2}^{\prime }R^{-1}X & Z_{2}^{\prime }R^{-1}Z_{1} & Z_{2}^{\prime }R^{-1}Z_{2}+V_{p}⊗I \end{array}\right]\left[\begin{array}{c} \hat{b}\\ \hat{a}\\ \hat{p} \end{array}\right]=\\ \left[\begin{array}{c} X^{\prime }R^{-1}y\\ Z_{1}^{\prime }R^{-1}y\\ Z_{2}^{\prime }R^{-1}y \end{array}\right], \end{array}$$

where **H** is the pedigree-based relationship matrix augmented with genomic information, **I** is the identity matrix, **R** is the diagonal matrix of heterogeneous residuals ranging from birth to 60 mo of age, **V**_g_ and **V**_p_ are 3 × 3 variance-covariance matrices for additive genetic and permanent environment effect, respectively, and ⊗ is the Kronecker product. The inverse of **H** was defined as

$$H^{-1}=A^{-1}+\left[\begin{array}{cc} 0 & 0\\ 0 & G^{-1}-A_{22}^{-1} \end{array}\right],$$

where **A**^−1^ is the inverted numerator relationship matrix, **G**^−1^ is the inverted genomic relationship matrix, and $A_{22}^{-1}$ is the inverted numerator relationship matrix of the genotyped individuals (Aguilar et al., 2010). The **G** matrix was calculated by the first method of VanRaden (2008) and blended with **A**_22_ as (1 − *α*)**G** + *α***A**_22_, where *α* = 0.05, to avoid a singularity problem. The means of diagonal and off-diagonal elements of **G** were adjusted to be equal to those of **A**_22_ (Chen et al., 2011). The variance structure of RRM was assumed as follows:

$$var\left[\begin{array}{c} a\\ p\\ e \end{array}\right]=\left[\begin{array}{ccc} V_{g}⊗H & 0 & 0\\ 0 & V_{p}⊗I & 0\\ 0 & 0 & R⊗I \end{array}\right].$$

Heritability estimates of height were obtained using the Gibbs sampling algorithm from the GIBBS3F90 program (Misztal et al., 2002), based on the same model described earlier but without genomic information. Solutions for GEBV were obtained by the preconditioned conjugate gradient method, and the convergence criterion was set to 10^−14^ in the squared ratio of the norm of the left-hand-side matrix of MME.

The ssGWAS approach (Wang et al., 2012) was applied to estimate SNP effects over time from the solutions of the random regression coefficients. In RRM-ssGWAS, computing *P*-values for a significance threshold is challenging because of the expensive computing cost to derive an inverted matrix on the left-hand side of the MME. However, the *P*-values can be computed by extending the proposed method of Aguilar et al. (2019). The algorithm included the following 3 steps (Campbell et al., 2019; Oliveira et al., 2019b). First, the GEBV of the *l*th individual at age *t* was calculated by ${\hat{GEBV}}_{l}=\Phi_{t}\hat{a_{l}}$ where $\Phi_{t}$ is the Legendre orthogonal polynomial matrix at age *t*. Second, the SNP effect at age *t* $\left({\hat{SNP}}_{t}\right)$ was obtained as $qW^{\prime }G^{-1}{\hat{GEBV}}_{l},$ where **W** is the marker matrix, *q* is the weighting factor of **G** calculated as $1/2\overset{m}{\underset{o=1}{\sum }}p_{o}\left(1-p_{o}\right),$ where *m* is the number of SNP and *p* is the allele frequency of *o*th SNP. In the last step, to obtain the *P*-values of SNP effects, we estimated their prediction error variances (**PEV**) by calculating $q^{2}{\left(1-\alpha \right)}^{2}W^{\prime }G^{-1}\left(G\sigma_{u_{t}}^{2}-C_{t}^{22}\right)GW,$ where $C_{t}^{22}$ is the inverted submatrix of PEV for genotyped animals at age *t* (Aguilar et al., 2019). Here, $C_{t}^{22}=\Phi_{t}C^{22}\Phi_{t}^{\prime },$ where **C**^22^ is the PEV matrix obtained by inverting the left-hand-side matrix in MME (Campbell et al., 2019). The sparse inversion technique implemented in the YAMS package (Masuda et al., 2014) was used to obtain **C**^22^. The *P*-values of SNP effects at age *t* were calculated by

$$2\left(1-\phi \left(\left|\frac{{\hat{SNP}}_{ot}}{std\left({\hat{SNP}}_{ot}\right)}\right|\right)\right),$$

where ϕ is the cumulative standard normal function.

Multiple testing corrections for GWAS were performed by inferring the effective number of SNP (Li and Ji, 2005), which is calculated based on the eigenvalues of the SNP correlation matrix, using the “poolr” R package (Cinar and Viechtbauer, 2022). The longitudinal ssGWAS was implemented using in-house software written in Fortran95.

The potential functional roles of genes that contained significant SNP or genes located upstream or downstream of the significant SNP (500,000 bp) were investigated further using Gene Ontology (**GO**) enrichment analysis. The nearby genes were queried from the Genome Ensemble database (release version 94) using the biomaRt Bioconductor package (Durinck et al., 2009) coupled with the UMD3.1 annotation. The GO enrichment analysis was performed using the “GOstats” Bioconductor package (Falcon and Gentleman, 2007).

The heritability estimates of height between birth and 60 mo of age ranged from 0.15 to 0.83, with an average of 0.66. The estimates were lower at earlier ages and then became constant at approximately 20 mo of age. Although we did not find any previous studies reporting genetic parameters of height using RRM, the increased heritability estimates at later ages were similar to those of BW derived from RRM in dairy and beef cattle (Arango et al., 2004; Yin and König, 2018). Manhattan plots of height at 6 age points (10, 20, 30, 40, 50, and 60) are presented in Figure 1. The solid blue lines denote the genome-wide threshold set to −log_10_[1 − (1 − 0.05)^1/^*^n^*] = 5.24, where *n* = 8,876 is the effective number of SNP. The RRM-ssGWAS analysis identified persistent SNP across age groups. However, transient SNP whose effects vary over time were not detected. One SNP, Hapmap45189-BTA-43948 (rs41636786), on BTA 18 was consistently significant across all ages (−log_10_ *P*-values ranging from 6.64 to 8.09). Similarly, Hapmap40537-BTA-43945 (rs41582522), a neighbor of Hapmap45189-BTA-43948, was identified as a significant SNP at 40, 50, and 60 mo of age (−log_10_ *P*-values in the range of 5.47 to 5.89). We also investigated the change of proportion of the total additive genetic variance explained by each marker over time. The results showed a similar trend with the *P*-value-based Manhattan plots. Namely, the peak was observed on BTA18, and Hapmap45189-BTA-43948 (rs41636786) always had the largest value in the range of 0.12 to 0.16% across the 6 time periods. Several markers on BTA18 close to the 2 identified SNP have also been reported in recent studies using Canadian Holstein bulls (Abo-Ismail et al., 2017) and large-scale meta-analyses (Bouwman et al., 2018).

**Figure 1:**
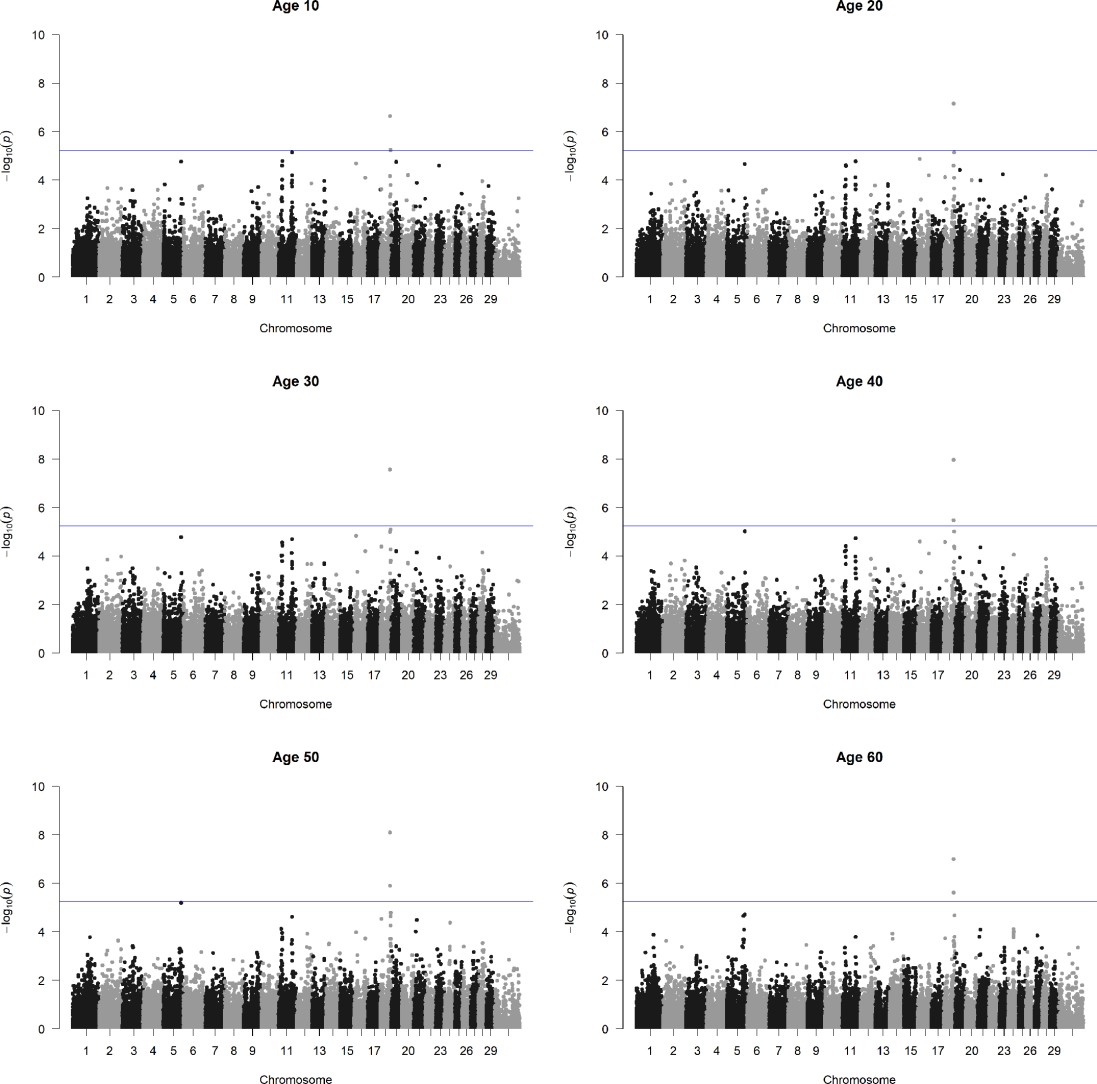
Manhattan plots of height at 10, 20, 30, 40, 50, and 60 mo of age. The solid blue lines denote the genome-wide threshold after multiple testing correction.

A total of 12 candidate genes were found in the vicinity of 2 significant SNP at 58.4 to 58.6 Mbp on BTA18 (Table 1). The identified genomic region in this study has been associated with some traits in previous studies. The zinc finger protein 613 (*ZNF613*) gene, which is one of the candidate genes identified in this study, has been reported to be associated with longevity (Zhang et al., 2016) and gestation length (Fang et al., 2019). Within the genomic region identified in this study, Müller et al. (2017) detected significant QTL on calving difficulty and fertility traits, and Maltecca et al. (2011) and Cole et al. (2014) found several SNP with a large effect on gestation length and birth weight, respectively. Cole et al. (2009) found a region associated with calving and body conformation traits, and hypothesized that the sialic acid binding Ig-like lectin 5 (*SIGLEC5*) gene located at 57.9 Mbp (based on the UMD 3.1 assembly) may increase calf size in association with longer gestation length. Height is known to have a strong genetic correlation with BW and body conformation traits, including body depth and rump width (Cole et al., 2009, 2014). In addition, larger animals have an increased risk of earlier culling, which is related to longevity (Hansen et al., 1999). The results of this study suggest that the genomic region identified on BTA18 may influence many economically important traits in dairy cattle in a complex manner. The enrichment analysis identified 6, 3, and 8 GO terms in the biological process, molecular function, and cellular component categories, respectively, at a significance level of 0.05 (Table 2). Overall, the GO terms enriched are linked to phosphatase, which plays a role in bone development and growth.

**Table 1:** List of identified candidate genes on chromosome 18 associated with height

Ensemble gene ID	Position (bp)		Gene name
	Start	End	
ENSBTAG00000040603	57,900,691	57,910,816	Zinc finger protein 175 (*ZNF175*)
ENSBTAG00000023365	57,927,152	57,929,923	
ENSBTAG00000045880	57,938,695	57,953,490	
ENSBTAG00000019227	57,997,837	58,000,711	
ENSBTAG00000038487	58,130,465	58,141,877	Zinc finger protein 613 (*ZNF613*)
ENSBTAG00000006954	58,151,318	58,157,607	Zinc finger protein 432 (*ZNF432*)
ENSBTAG00000003541	58,180,500	58,183,055	Zinc finger protein 614 (*ZNF614*)
ENSBTAG00000010046	58,212,122	58,220,103	Zinc finger protein 350 (*ZNF350*)
ENSBTAG00000019851	58,425,828	58,448,787	Protein phosphatase 2 scaffold subunit A alpha (*PPP2R1A*)
ENSBTAG00000018162	58,494,237	58,495,142	
ENSBTAG00000047617	58,571,817	58,572,767	
ENSBTAG00000047791	58,617,315	58,618,271

**Table 2:** List of Gene Ontology (GO) terms associated with height

Category	ID	*P*-value	GO term
Biological process	GO:0043666	0.006	Regulation of phosphoprotein phosphatase activity
	GO:0035304	0.007	Regulation of protein dephosphorylation
	GO:0010921	0.008	Regulation of phosphatase activity
	GO:0035303	0.009	Regulation of dephosphorylation
	GO:0007059	0.016	Chromosome segregation
	GO:0006470	0.017	Protein dephosphorylation
	GO:0016311	0.025	Dephosphorylation
Molecular function	GO:0019888	0.003	Protein phosphatase regulator activity
	GO:0019208	0.003	Phosphatase regulator activity
	GO:0030234	0.047	Enzyme regulator activity
Cellular component	GO:0016328	0.003	Lateral plasma membrane
	GO:0000775	0.012	Chromosome, centromeric region
	GO:0030425	0.015	Dendrite
	GO:0097447	0.015	Dendritic tree
	GO:0098687	0.017	Chromosomal region
	GO:0036477	0.023	Somatodendritic compartment
	GO:0043005	0.049	Neuron projection
	GO:0005694	0.050	Chromosome

In this study, we applied the RRM-ssGWAS to longitudinal height data. The ssGWAS approach enables the simultaneous inclusion of data from phenotyped, but nongenotyped, animals in the analyses. In addition, it can identify significant SNP based on their *P*-values as shown in this study. However, computation of *P*-values in RRM-ssGWAS may be challenging when using a larger left-hand side matrix coupled with higher-order random regression coefficients and more genotyped animals because obtaining the inverse matrix is difficult. Therefore, in such a situation, different longitudinal GWAS approaches (Ning et al., 2017, 2018; Du et al., 2021) may be more suitable.

To the best of our knowledge, this is the first longitudinal study to investigate the changes in genomic marker effects over time using longitudinal height data in dairy cattle. Although the genetic correlations of height across the 6 age points were not constant and ranged between 0.39 and 0.99, the ssGWAS results did not identify significant transient genes affecting height at only one of these periods. The results of this study suggest that many genes with minor effects influence height over time. For the growth trait of BW, Yin and König (2019) found significant persistent genes at birth, 2–3 mo, and 13–14 mo of age, with low-to-moderate estimates of genetic correlations among them. As reported by Yin and König (2019), the most significant candidate genes for growth traits may have pleiotropic effects at different age points.

This study investigated the locations of QTL regions for dairy cattle height using time-series data by integrating ssGWAS and RRM. The longitudinal ssGWAS estimates SNP effects while accounting for covariance across age groups. In conclusion, we detected a single persistent QTL region on BTA18, likely to continuously influence height across growth stages and have a pleiotropic effect on various traits in dairy cattle. Therefore, further analysis is warranted to understand the association with important traits, such as calving, reproduction, and body conformation traits in Japanese Holsteins. In addition, investigating the effect of the identified candidate genes on heifer growth rate is expected to offer further insights into gene function. This study serves as the first step toward understanding the genetic basis of height in Japanese Holstein dairy cattle.
